# Feeding-induced rearrangement of green leaf volatiles reduces moth oviposition

**DOI:** 10.7554/eLife.00421

**Published:** 2013-05-14

**Authors:** Silke Allmann, Anna Späthe, Sonja Bisch-Knaden, Mario Kallenbach, Andreas Reinecke, Silke Sachse, Ian T Baldwin, Bill S Hansson

**Affiliations:** 1 Department of Molecular Ecology, Max Planck Institute for Chemical Ecology, Jena, Germany; 2 Department of Evolutionary Neuroethology, Max Planck Institute for Chemical Ecology, Jena, Germany; Wageningen University, The Netherlands

**Keywords:** *Manduca sexta*, plant volatiles, oviposition, Ca imaging, *Datura wrightii*, Other

## Abstract

The ability to decrypt volatile plant signals is essential if herbivorous insects are to optimize their choice of host plants for their offspring. Green leaf volatiles (GLVs) constitute a widespread group of defensive plant volatiles that convey a herbivory-specific message via their isomeric composition: feeding of the tobacco hornworm *Manduca sexta* converts (*Z*)-3- to (*E*)-2-GLVs thereby attracting predatory insects. Here we show that this isomer-coded message is monitored by ovipositing *M. sexta* females. We detected the isomeric shift in the host plant *Datura wrightii* and performed functional imaging in the primary olfactory center of *M. sexta* females with GLV structural isomers. We identified two isomer-specific regions responding to either (*Z*)-3- or (*E*)-2-hexenyl acetate. Field experiments demonstrated that ovipositing *Manduca* moths preferred (*Z*)-3-perfumed *D. wrightii* over (*E*)-2-perfumed plants. These results show that (*E*)-2-GLVs and/or specific (*Z*)-3/(*E*)-2-ratios provide information regarding host plant attack by conspecifics that ovipositing hawkmoths use for host plant selection.

**DOI:** http://dx.doi.org/10.7554/eLife.00421.001

## eLife digest

Plants have developed a variety of strategies to defend themselves against herbivorous animals, particularly insects. In addition to mechanical defences such as thorns and spines, plants also produce compounds known as secondary metabolites that keep insects and other herbivores at bay by acting as repellents or toxins. Some of these metabolites are produced on a continuous basis by plants, whereas others—notably compounds called green-leaf volatiles—are only produced once the plant has been attacked. Green-leaf volatiles—which are also responsible for the smell of freshly cut grass—have been observed to provide plants with both direct protection, by inhibiting or repelling herbivores, and indirect protection, by attracting predators of the herbivores themselves.

The hawkmoth *Manduca sexta* lays its eggs on various plants, including tobacco plants and sacred Datura plants. Once the eggs have hatched into caterpillars, they start eating the leaves of their host plant, and if present in large numbers, these caterpillars can quickly defoliate and destroy it. In an effort to defend itself, the host plant releases green-leaf volatiles to attract various species of *Geocoris*, and these bugs eat the eggs.

One of the green-leaf volatiles released by tobacco plants is known as (*Z*)-3-hexenal, but enzymes released by *M. sexta* caterpillars change some of these molecules into (*E*)-2-hexenal, which has the same chemical formula but a different structure. The resulting changes in the ‘volatile profile’ alerts *Geocoris* bugs to the presence of *M. sexta* eggs and caterpillars on the plant.

Now Allmann et al. show that adult female *M. sexta* moths can also detect similar changes in the volatile profile emitted by sacred Datura plants that have been damaged by *M. sexta* caterpillars. This alerts the moths to the fact that *Geocoris* bugs are likely to be attacking eggs and caterpillars on the plant, or on their way to the plant, so they lay their eggs on other plants. This reduces competition for resources and also reduces the risk of newly laid eggs being eaten by predators. Allmann et al. also identified the neural mechanism that allows moths to detect changes in the volatile profile of plants—the *E*- and *Z*- odours lead to different activation patterns in the moth brain.

**DOI:** http://dx.doi.org/10.7554/eLife.00421.002

## Introduction

Insects rely on olfaction in most aspects of life: volatile signals guide them to food sources, mating partners and oviposition hosts. Especially for herbivorous insects, plant volatiles provide important cues to locate and identify appropriate host plants for their offspring. Upon herbivory, plants respond with an increased release and de novo synthesis of several volatile compounds from their vegetative tissues (Mumm and Dicke, 2010). These so-called herbivore induced plant volatiles can provide significant information to the surrounding environment as composition and abundance reflect several biotic and abiotic factors (Takabayashi et al., 1995; De Moraes et al., 1998; Gouinguené et al., 2001; Schuman et al., 2009; Hare, 2010).

Due to the context dependent composition of plant volatile signals, the ability to detect and discriminate volatile compounds is crucial for insects to generate appropriate behavioral responses. In insects and more specifically in the hawkmoth *Manduca sexta* (Lepidoptera/Sphingidae), olfactory sensory neurons (OSNs) located on the antennae detect odorant molecules (Kalinová et al., 2001; Shields and Hildebrand, 2001; Fraser et al., 2003; Spaethe et al., 2013) and convey this information to the antennal lobe (AL), the first olfactory processing center. The AL of *M. sexta* females consists of about 70 structural and functional subunits called olfactory glomeruli (Grosse-Wilde et al., 2011). OSNs expressing the same receptor, and thus responding to the same set of odorants, converge onto the same glomerulus in the AL (Gao et al., 2000; Vosshall, 2000) as has been demonstrated for *Drosophila melanogaster* and indirectly also in several moth species (Hansson, 1997). Spatio-temporal patterns of neuronal activity representing sensory input to the AL can be visualized by optical imaging methods (Hansson et al., 2003; Skiri et al., 2004; Carlsson et al., 2005; Silbering and Galizia, 2007) enabling identification of compound- and blend-specific responses in the AL of *M. sexta* (Hansson et al., 2003; Bisch-Knaden et al., 2012; Kuebler et al., 2012).

Green leaf volatiles (GLVs) constitute a large group of herbivore-induced plant volatiles characterized by a C6-backbone. While emitted only in trace amounts from healthy, undamaged plant tissue, they are emitted instantly after cell disruption (Turlings et al., 1995; D’Auria et al., 2007). GLVs are generated from C18-fatty acids via the enzymes lipoxygenase (LOX) and hydroperoxide lyase (HPL; Allmann et al., 2010). One of the most abundant GLVs, (*Z)*-3-hexenal, originates from the cleavage of α-linolenic acid through the activity of HPL and it partly rearranges to (*E)*-2-hexenal. Both alkenals can be further metabolized by an alcohol dehydrogenase (ADH) and alcohol acyltransferase (AAT; D’Auria et al., 2007) to the corresponding alcohols and their esters (Matsui, 2006).

GLVs have been assigned various plant defense-associated functions by directly inhibiting phytopathogens (Hamilton-Kemp et al., 1992; Nakamura and Hatanaka, 2002; Prost et al., 2005) and repelling several herbivore species (De Moraes et al., 2001; Kessler and Baldwin, 2001; Vancanneyt et al., 2001; Zhang and Schlyter, 2004). Remarkably, GLVs also function as indirect plant defenses by attracting foraging predators and host-seeking parasitoids to the plant and its attacker (Kessler and Baldwin, 2001; Shiojiri et al., 2006; Halitschke et al., 2008; Schuman et al., 2012) reminiscent of the role of other herbivore induced plant volatiles.

Due to their ubiquity and instant release, GLVs are thought to act as nonspecific signals of plant damage (Hatanaka et al., 1987; Hoballah et al., 2002). We recently showed that an enzymatic component of the oral secretions (OS) of *M. sexta* larvae adds an herbivory-specific feature to the GLV signal. Mechanically damaged leaves of *Nicotiana attenuata* released large amounts of (*Z*)-3-GLVs and low amounts of (*E*)-2-GLVs. However, when the plant was attacked by *M. sexta* caterpillars or when puncture wounds of plant leaves were treated with *M. sexta’s* OS, the amount of (*E*)-2-GLVs released increased, while the amount of (*Z*)-3-GLVs decreased, resulting in a distinct change in the (*Z*)-3/(*E*)-2-ratio of GLV emissions. This herbivore-induced change in the (*Z*)-3/(*E*)-2-ratio attracted the generalist hemipteran predator *Geocoris* spp., which decreased the herbivore load on the plant by feeding on herbivore eggs (Allmann and Baldwin, 2010).

Our discovery of a (3*Z*):(2*E*)-enal isomerase in the OS of *M. sexta* larvae raises many questions. Why does *Manduca* produce an enzyme that generates volatiles which betray the insect to its enemies, and why did evolution not select against this isomerase? The enzyme might be maladaptive and therefore is, or will be, under negative selection. The occurrence of this specific isomerase activity in at least two other lepidopteran species (Allmann and Baldwin, 2010) however, suggests that it may have a beneficial function that outweighs the larva’s net costs of maintaining such an enzyme. It is well known that plants exchange information above ground by releasing volatiles into the air (Baldwin, 2010), which can be perceived by insects as well. Insects can use plant derived volatiles for communication by giving the herbivore induced volatile blend a ‘personal’ note—in our case, by converting (*Z*)-3-GLVs to their structural isomers and by changing the (*Z*)-3/(*E*)-2 ratio. Which message could *M. sexta* larvae thereby communicate? In this study we hypothesized that the altered GLV emission might serve to reduce the number of competitors on their host plant by informing conspecific ovipositing moths that this plant is already occupied and, possibly, receiving increased predation. Reduced oviposition of *Manduca* moths in response to feeding damage has been shown in field experiments with *M. quinquemaculata* (Kessler and Baldwin, 2001) as well as under laboratory conditions with *M. sexta* (Spaethe et al., 2013). Deviating from the previous study, we chose *Datura wrightii* (Solanaceae) for our experiments. *Datura* is a highly preferred host plant of both *M. sexta* and the congeneric *M. quinquemaculata* for both nectar feeding (Alarcón et al., 2008; Kessler, 2012) and oviposition (Spaethe et al., 2013). Its distribution covers southwestern USA (Avery, 1959; Munz, 1973) overlapping with the occurrence of both *Manduca* species. The perennial shrub is repeatedly described to quickly regrow leaves after herbivore damage (Bronstein et al., 2009; Reisenman et al., 2010, 2013). Laboratory experiments failed to find reduced oviposition on damaged *D. wrightii* (Reisenman et al., 2013; Spaethe et al., 2013) suggesting flexibility in oviposition choice of *Manduca* females. As the previously examined *N. attenuata* (Gaquerel et al., 2009)*, D. wrightii,* respond to *Manduca* herbivore attack by emitting GLVs (Hare and Sun, 2011). While we investigated GLV emission during the day when focusing on the diurnal egg predator *Geocoris* ssp., *Manduca* moths oviposit at twilight and night (Madden and Chamberlin, 1945; Lingren et al., 1977). Therefore, we decided to collect volatiles during these times instead. We expected the shift to occur also during the night, as in several plant species GLV emission has been shown to occur also in the dark period (Loughrin et al., 1994; Arimura et al., 2008), and the respective shift in the (*Z*)-3/(*E*)-2-ratio is caused by *M. sexta* oral secretions and not by the plant itself (Allmann and Baldwin, 2010). However, volatile emissions vary with light regime (Halitschke et al., 2000; De Moraes et al., 2001; Gouinguene and Turlings, 2002; Morker and Roberts, 2011), and we therefore chose two nocturnal light conditions differing by moonlight intensity to examine whether light intensity affects GLV emission in *D. wrightii*. We performed functional imaging in the antennal lobe of female *M. sexta* moths asking whether (*Z*)-3- and (*E*)-2-structural isomers of any of the tested GLVs can be discriminated by the olfactory system. In classical host recognition experiments the Colorado potato beetle *Leptinotarsa decemlineata* has been shown to recognize and avoid altered ratios of (*Z*)-3- and (*E*)-2-GLVs emitted by its host plant *Solanum tuberosum* (Visser and Avé, 1978). Furthermore, enantioselectivity has been reported for projection neurons in the female AL of *M. sexta* in response to (+)- and (−)-linalool (Reisenman et al., 2004). Thus, we hypothesized that *M. sexta* females would be able to differentiate between (*Z*)-3 and (*E*)-2-isomers of at least one GLV. If so, ovipositing *M. sexta* should avoid plants with increased levels of (*E*)-2-GLVs as they indicate host plants with increased larval feeding competition and predation risk (Allmann and Baldwin, 2010). Here we show by combining field studies with neurophysiological imaging techniques that (i) OS-induced *D. wrightii* plants have altered (*Z*)-3/(*E*)-2-ratios also during the night under both laboratory and field conditions, (ii) *Manduca* females detect and discriminate the (*Z*)-3- and (*E*)-2-isomers and (iii) show ovipositional preference for high (*Z*)-3/(*E*)-2-GLV ratios.

## Results

### Application of *M. sexta* OS to leaf wounds triggers pronounced changes in the GLV profile of *Datura wrightii*

**Figure 1.**
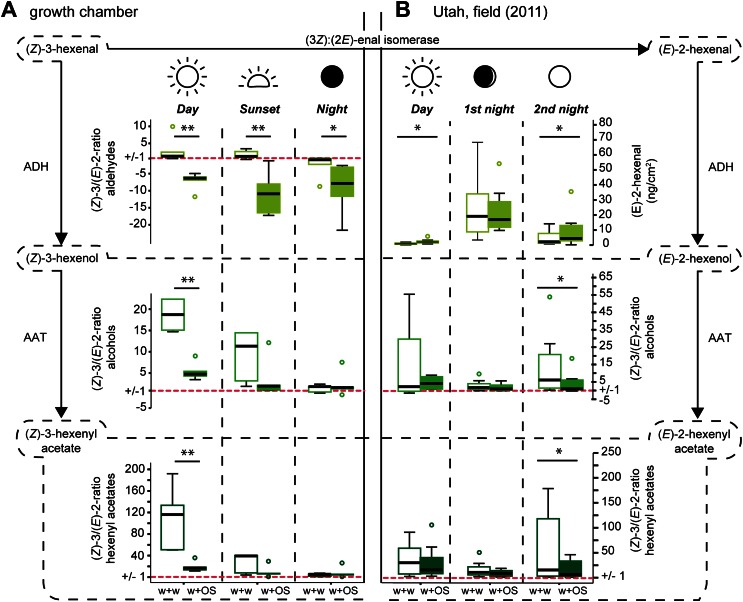
Diurnal changes in the emitted (*Z*)-3/(*E*)-2-ratios of GLVs in *Datura wrightii* plants. (*Z*)-3/(*E*)-2-ratios of GLVs in *Datura wrightii* plants represented as box plots. (**A**) Growth chamber experiment: a single not yet fully developed leaf of each *D. wrightii* plant was mechanically wounded and treated with water (w + w) or *M. sexta* OS (w + OS) during three different light conditions to mimic day, sunset, and night. (**B**) Field experiment: Three single previously undamaged leaves per plant were chosen and randomly assigned to a treatment (control, w + w or w + OS). Values of the control leaf were subtracted from the values of treated leaves. As (*Z*)-3-hexenal was not detectable in any of the field samples (*E*)-2-hexenal values are displayed in ng*cm^−2^*2h^−1^ (adsorbents used in field collection are not accountable for the absence of (*Z*)-3-hexenal; Table 6). For visual simplifications (*Z*)-3/(*E*)-2-ratios <1 are represented as their negative reciprocal. Values above ‘1’ (red dotted line) thus represent treatment-groups that produced more of the (*Z*)-3-isomer and values below ‘1’ represent treatment-groups that produced more of the (*E*)-2-isomer. Asterisks indicate significant differences between treatments (**A**: Mann–Whitney U test, **p≤0.01, *p≤0.05; n = 5), (**B**: Wilcoxon signed-rank test, *p<0.05; n = 8). ADH: alcohol dehydrogenase; AAT: alcohol acyl-transferase. The median is represented as a line in each box, box outlines mark the 25% and 75% percentiles; outliers are depicted as circles (if value > 1.5× the interquartile range). For raw data, see F1AB_AllmannSpaethe2012_volatiles.xlsx (Dryad: Allmann et al., 2012). **DOI:** http://dx.doi.org/10.7554/eLife.00421.003

To investigate whether application of *M. sexta’s* OS onto wounded leaves of *Datura wrightii* plants causes a similar shift in the (*Z*)-3/(*E*)-2-ratio as observed in *Nicotiana attenuata*, we compared the emissions of mechanically wounded *D. wrightii* plants that were treated with either water as a control (w + w) or with *M. sexta’s* OS (w + OS) in growth chamber experiments. During the day, application of OS onto wounds caused a significant decrease in the (*Z*)-3/(*E*)-2-ratio of the GLVs released from *Datura* plants compared with control plants (Figure 1A, day).

**Figure 2.**
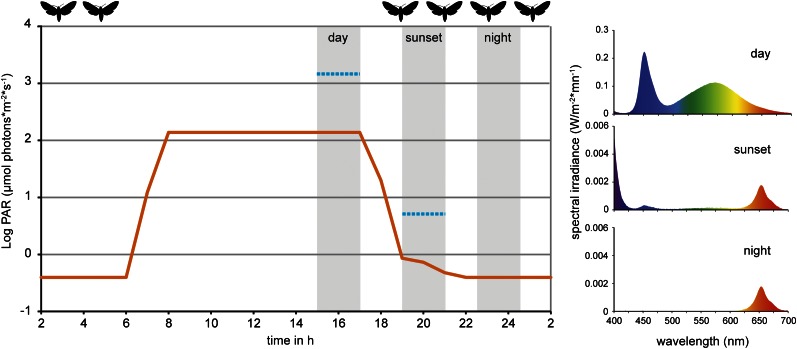
Light conditions during laboratory volatile collection. Light composition and intensity changed within 24 hr to simulate day, sunset and night condition. Photosynthetically active radiation (PAR, μmol photons*m^−2^*s^−1^, orange line) was measured for every light composition and ranged from 0.39 ± 0.01 SE at night to 138.37 ± 0.09 SE at full day conditions. Blue lines denote PAR values measured in the field during the respective volatile collection event (during the night samplings, PAR was below detection limit). For the graph values were logarithmized. Grey areas denote volatile collection events; respective light spectra are shown on the right. For representational reasons time scale starts at 2 am. Flight activity, related to nectar feeding and oviposition (Madden and Chamberlin, 1945; Lingren et al., 1977), is indicated on top of the graph. For raw data, see F2_AllmannSpaethe2012_light.xlsx (Dryad: Allmann et al., 2012). **DOI:** http://dx.doi.org/10.7554/eLife.00421.004

**Figure 3.**
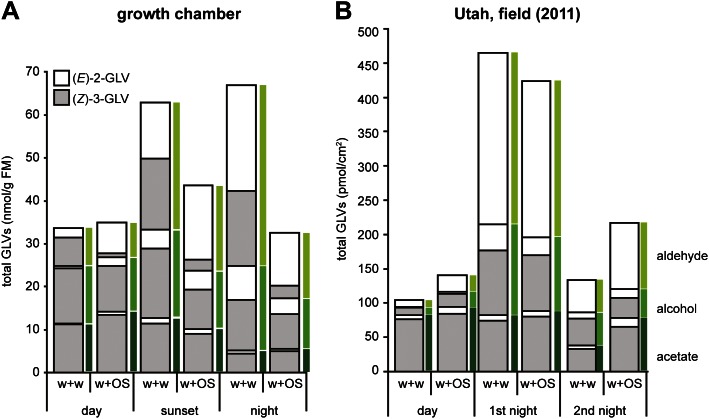
Total amounts of GLVs released from *Datura wrightii* plants at different times of the day in laboratory and field experiments. Mean release of major GLVs from *Datura wrightii* plants at different times of the day and at different light intensities. Grey and white bars represent (*Z*)-3- and (*E*)-2-GLVs, respectively. Single leaves were mechanically damaged and volatiles were trapped for 2 hr immediately after wounds had been treated with either water (w + w) or with *M. sexta’s* OS (w + OS). (**A**) GLV emissions of *D. wrightii* plants under controlled light conditions in a growth chamber. Light conditions are explained in this figure. Quantities are given in nmol/g fresh mass (FM)/2 hr; n = 5. (**B**) GLV emissions of *D. wrightii* plants naturally grown in the field. Quantities are given in pmol/cm^2^/2 hr; n = 8. For an approximate comparison between (**A**) and (**B**): 50 cm^2^ leaf area ≈ 1 g FM. Colored bars mark the emission of aldehydes (light green), alcohols (green) and acetates (dark green). For raw data, see F1AB_AllmannSpaethe2012_volatiles.xlsx (Dryad: Allmann et al., 2012). **DOI:** http://dx.doi.org/10.7554/eLife.00421.005

Since *Manduca* moths are crepuscular and nocturnal insects (Theobald et al., 2010), we repeated the experiment under low light and no-light conditions to mimic sunset and night (Figure 2). The (*Z*)-3/(*E*)-2-ratio of the aldehydes differed significantly between treatments also at sunset and night light intensities (Figure 1A, sunset). However, the (*Z*)-3/(*E*)-2-ratio of w + w treated plants also decreased with decreasing light intensities, which was mainly caused by increased (*E*)-2-hexenal emissions (Figure 3 and Table 1). Correspondingly, treatment-dependent differences in (*Z*)-3/(*E*)-2-ratios for the alcohol and the hexenyl acetate decreased under lower light conditions and were not found during the night (Figure 1A, sunset, night).

**Table 1.** GLV emission of *Datura wrightii* plants in the growth chamber during the first 2 hr after w + w or w + OS treatment with 100% light (day), 20–10% light (sunset) or 0% light (night) **DOI:** http://dx.doi.org/10.7554/eLife.00421.006

	Class	Common name	RT	*volatile release in µg / g leaf fresh mass*	
				w + w	w + OS
Day	Aldehyde	*(Z)*-3-hexenal	8.54	0.64 ± 0.293	0.097 ± 0.027
		*(E)*-2-hexenal	10.49	0.22 ± 0.109	0.7 ± 0.17
	Alcohol	*(Z)*-3-hexenol	14.98	1.30 ± 0.511	1.06 ± 0.275
		*(E)*-2-hexenol	15.57	0.058 ± 0.034	0.195 ± 0.034
	Hexenylester	*(Z)*-3-hexenyl acetate	13.28	1.59 ± 0.442	1.92 ± 0.244
		*(E)*-2-hexenyl acetate	13.75	0.017 ± 0.004	0.105 ± 0.018
		*(Z)*-3-hexenyl butyrate	17.07	0.028 ± 0.009	0.051 ± 0.016
		*(E)*-2-hexenyl butyrate	17.44	0.01 ± 0.002	0.017 ± 0.004
Sunset	Aldehyde	*(Z)*-3-hexenal	8.54	1.62 ± 0.5	0.26 ± 0.118
		*(E)*-2-hexenal	10.49	1.28 ± 0.775	1.69 ± 0.697
	Alcohol	*(Z)*-3-hexenol	14.98	1.62 ± 0.433	0.93 ± 0.308
		*(E)*-2-hexenol	15.57	0.45 ± 0.315	0.44 ± 0.183
	Hexenylester	*(Z)*-3-hexenyl acetate	13.28	1.62 ± 0.431	1.28 ± 0.511
		*(E)*-2-hexenyl acetate	13.75	0.18 ± 0.12	0.158 ± 0.067
		*(Z)*-3-hexenyl butyrate	17.07	0.039 ± 0.011	0.031 ± 0.003
		*(E)*-2-hexenyl butyrate	17.44	0.013 ± 0.004	0.01 ± 0.001
Night	Aldehyde	*(Z)*-3-hexenal	8.54	1.71 ± 0.732	0.28 ± 0.118
		*(E)*-2-hexenal	10.49	2.43 ± 0.521	1.22 ± 0.697
	Alcohol	*(Z)*-3-hexenol	14.98	1.18 ± 0.35	0.81 ± 0.308
		*(E)*-2-hexenol	15.57	0.79 ± 0.14	0.37 ± 0.183
	Hexenylester	*(Z)*-3-hexenyl acetate	13.28	0.63 ± 0.268	0.71 ± 0.511
		*(E)*-2-hexenyl acetate	13.75	0.093 ± 0.04	0.083 ± 0.067
		*(Z)*-3-hexenyl butyrate	17.07	0.036 ± 0.002	0.033 ± 0.003
		*(E)*-2-hexenyl butyrate	17.44	0.01 ± 0.002	0.014 ± 0.001

Mean (±SEM; n = 5) release of GLVs in *D. wrightii* plants. A single not yet fully developed leaf of each plant was mechanically wounded and treated with water (w + w) or *M. sexta* OS (w + OS) during the day (**A**, 100% light), sunset (**B**, 20–10% light) and night (**C**, 0% light). Volatiles are listed by chemical classes and in order of their retention time.

To evaluate whether w + w and w + OS treated plants release GLVs in distinguishable ratios under normally variable conditions found in nature, we trapped volatiles during daylight and repeatedly at night from a native *D. wrightii* population in the Utah desert during the 2011 field season. We performed the experiments on two different days using eight plants for each sampling. Three equally sized leaves of each plant were selected and randomly assigned to one of the treatments (control, w + w or w + OS). Similar to previous experiments with *N. attenuata* (Allmann and Baldwin, 2010) we were unable to detect (*Z*)-3-hexenal in any of the samples.

During the day the application of OS to the wounds caused a significant increase in (*E*)-2-hexenal emissions compared with w+w treated leaves (Figure 1B, day, and Table 2, day). As seen from the climate chamber experiment, average (*Z*)-3/(*E*)-2-ratio of the hexenyl acetates decreased (Figure 1B, day), but this change was not significant.

**Table 2.** GLV emission of native *Datura wrightii* plants in the field (2011) during the first 2 hr after w + w or w + OS treatment; during day (1:30–3:30 pm), first or second night (0–2 am) **DOI:** http://dx.doi.org/10.7554/eLife.00421.007

	Class	Common name	RT	Volatile release in ng/cm^2^ leaf		
Control	w + w	w + OS
Day	Aldehyde	*(E)*-2-hexenal	10.87	0.062 ± 0.006	1.02 ± 0.233	2.43 ± 0.597
	Alcohol	*(Z)*-3-hexenol	15.38	0.137 ± 0.067	1.21 ± 0.28	2.07 ± 0.465
		*(E)*-2-hexenol	15.97	0.248 ± 0.035	0.368 ± 0.088	0.57 ± 0.148
	Hexenylester	*(Z)*-3-hexenyl acetate	13.66	0.26 ± 0.083	11.1 ± 1.881	12.3 ± 2.067
		*(E)*-2-hexenyl acetate	14.13	0.01 ± 0.002	0.87 ± 0.396	1.34 ± 0.564
		*(Z)*-3-hexenyl butyrate	17.44	0.011 ± 0.002	0.22 ± 0.181	0.19 ± 0.142
		*(E)*-2-hexenyl butyrate	17.8	0.004 ± 0.001	0.007 ± 0.002	0.006 ± 0.001
First night	Aldehyde	*(E)*-2-hexenal	10.87	0.103 ± 0.013	24.6 ± 7.844	22.5 ± 5.312
	Alcohol	*(Z)*-3-hexenol	15.38	0.032 ± 0.006	9.6 ± 2.028	8.2 ± 3.734
		*(E)*-2-hexenol	15.97	0.296 ± 0.023	4.12 ± 0.955	2.89 ± 0.855
	Hexenylester	*(Z)*-3-hexenyl acetate	13.66	0.165 ± 0.028	10.7 ± 3.621	11.53 ± 4.291
		*(E)*-2-hexenyl acetate	14.13	0.009 ± 0.001	1.14 ± 0.371	1.15 ± 0.306
		*(Z)*-3-hexenyl butyrate	17.44	0.007 ± 0.001	0.022 ± 0.008	0.04 ± 0.022
		*(E)*-2-hexenyl butyrate	17.8	0.002 ± 0	0.006 ± 0.002	0.007 ± 0.003
Second night	Aldehyde	*(E)*-2-hexenal	10.87	0.055 ± 0.009	4.7 ± 1.877	9.5 ± 4.009
	Alcohol	*(Z)*-3-hexenol	15.38	0.034 ± 0.018	4.0 ± 1.225	2.94 ± 0.522
		*(E)*-2-hexenol	15.97	0.177 ± 0.021	0.99 ± 0.427	1.47 ± 0.554
	Hexenylester	*(Z)*-3-hexenyl acetate	13.66	0.089 ± 0.024	4.8 ± 2.114	9.4 ± 4.708
		*(E)*-2-hexenyl acetate	14.13	0.01 ± 0.002	0.74 ± 0.505	1.77 ± 0.972
		*(Z)*-3-hexenyl butyrate	17.44	bld.	0.032 ± 0.019	0.039 ± 0.013
		*(E)*-2-hexenyl butyrate	17.8	bld.	0.005 ± 0.003	0.007 ± 0.002

Mean (±SEM; n = 5) release of GLVs in *D. wrightii* plants in nature. A single not yet fully developed leaf of each plant was mechanically wounded and treated with water (w + w) or *M. sexta* OS (w + OS) during the day (**A**, 1:30–3:30 pm) and during night (**B**, first night, **C**, second night, 0–2 am). Volatiles are listed by chemical classes and in order of their retention time; bld.: below the limit of detection.

During the first night-experiment (first night, average temperature 17.6 ± 0.7°C, wind speed 1.1 ± 0.8 m/s, waxing crescent lunar illumination with 9% of the moon illuminated), plants of both treatments released very high but similar amounts of (*E*)-2-hexenal, and the (*Z*)-3/(*E*)-2-ratios of the alcohols and hexenyl acetates were low, but did not differ between treatments, resembling the results of the night trapping in the growth chamber (Figure 1B, first night, and Table 2, first night).

During the second experiment (second night; average temperature 24.6 ± 0.8°C, wind speed 0.7 ± 0.8 m/s, full moon), approximately 2 weeks later, w + OS-treated plants released significantly higher amounts of (*E*)-2-hexenal (twofold increase compared with w + w treated plants) and the (*Z*)-3/(*E*)-2-ratios of the hexenols and hexenyl acetates were significantly lower compared with mechanically wounded plants that were treated with water only (Figure 1B, second night).

### (*Z*)-3- and (*E*)-2-GLVs evoke different activation patterns in the antennal lobes of *Manduca sexta*

**Figure 4.**
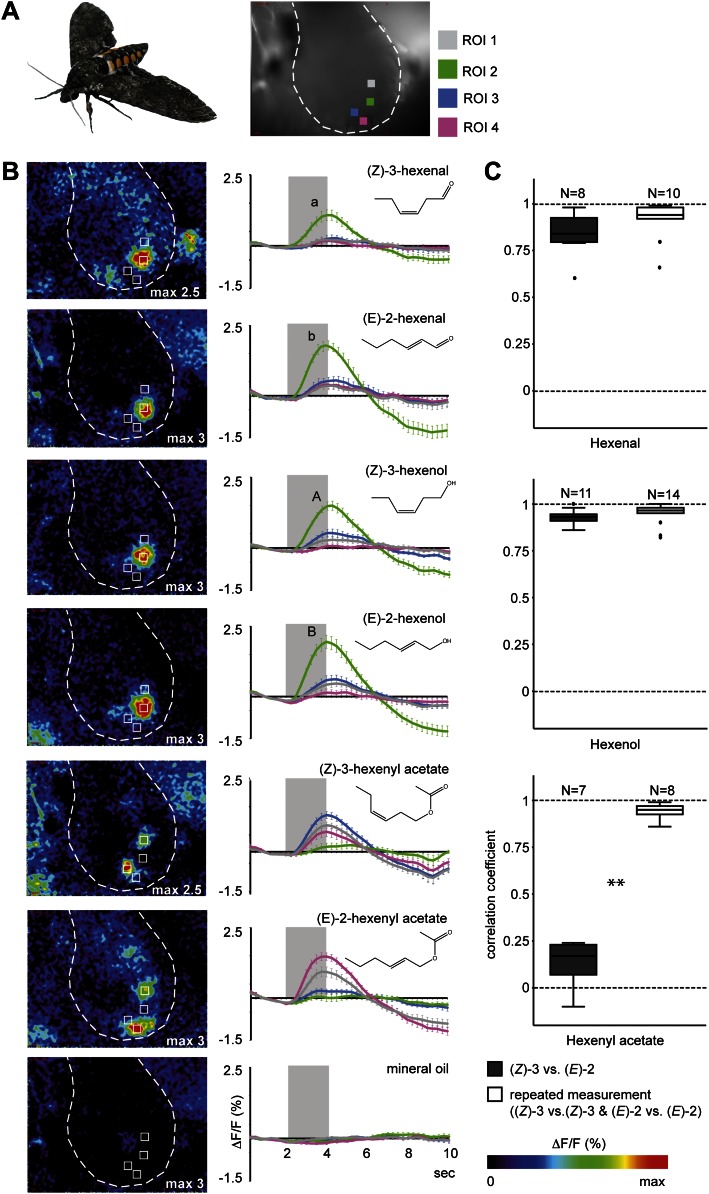
Calcium activity patterns of the (*Z*)-3- and (*E*)-2-isomers in the *M. sexta* antennal lobe (AL). (**A**) View onto the AL (marked by outline) of a *Manduca sexta* female after bath application with the calcium-sensitive dye calcium-green-AM. Stimulations with the six tested GLVs resulted in the activation of four regions in the AL most probably corresponding to single glomeruli (four ROIs, regions of interest). (**B**) Representative false color-coded images show calcium responses in the AL after odor stimulation. Images are individually scaled to the strongest activation (given by the max value in each image). Time traces show activity of ROI 1, 2, 3 and 4 (n = 10) in response to odor stimulation (2 s; grey bar). Error bars represent standard errors of means. For hexenal and hexenol, stimulations with the (*E*)-2-isomers activated ROI 2 significantly stronger than did stimulations with the (*Z*)-3-isomers (Wilcoxon signed-rank test: hexenal: p<0.01, hexenol: p<0.05). ΔF: change in fluorescence; F: background fluorescence. For raw data, see F4B_AllmannSpaethe2012_timetracesGlvs.xlsx (Dryad: Allmann et al., 2012). (**C**) Comparison of response pattern similarity for repeated stimulations of one structural isomer ((*Z*)-3 vs (*Z*)-3 Or (*E*)-2 vs (*E*)-2, white boxes) and for both structural isomers ((*E*)-2 vs (*Z*)-3, grey boxes); sample size is given above the boxes (Mann–Whitney U test: hexenal: p>0.05; hexenol: p>0.05, hexenyl acetate: p<0.001). For raw data, see F4C_AllmannSpaethe2012_correlationcoefficientsGlvs.xlsx (Dryad: Allmann et al., 2012). **DOI:** http://dx.doi.org/10.7554/eLife.00421.008

To evaluate if female *M. sexta* moths are physiologically able to discriminate between (*Z*)-3- and (*E*)-2-GLVs and between different (*Z*)-3/(*E*)-2-ratios we performed functional calcium imaging in the antennal lobes (AL) of females. Odor-evoked calcium changes in response to exposure to the pure (*E*)-2- and (*Z*)-3-isomers of hexenal, hexenol and hexenyl acetate led to activity in discrete regions corresponding to specific glomeruli in the AL of *M. sexta* females (Figure 4A,B). Aldehyde and alcohol structural isomers activated one single specific region (region of interest 2 [ROI 2], green), with significantly stronger responses to the (*E*)-2- compared with (*Z*)-3-isomers (Figure 4B). (*Z*)-3-hexenyl acetate and its structural isomer activated three different regions in the female AL: a significantly (*Z*)-3-specific (ROI 3, blue), a significantly (*E*)-2-specific (ROI 4, pink) and an isomer-unspecific region (ROI 1, grey, Figure 4B). The differences in activation patterns caused by stimulations with (*Z*)-3- or (*E*)-2-hexenyl acetate (Figure 4C) strongly suggest that the two odors activated OSNs expressing different sets of odorant receptor types on the female antennae. Of all tested GLVs, hexenyl acetate was the only compound eliciting isomer-specific responses in the AL, therefore we focused on (*Z*)-3- and (*E*)-2-hexenyl acetate for all further experiments.

**Figure 5.**
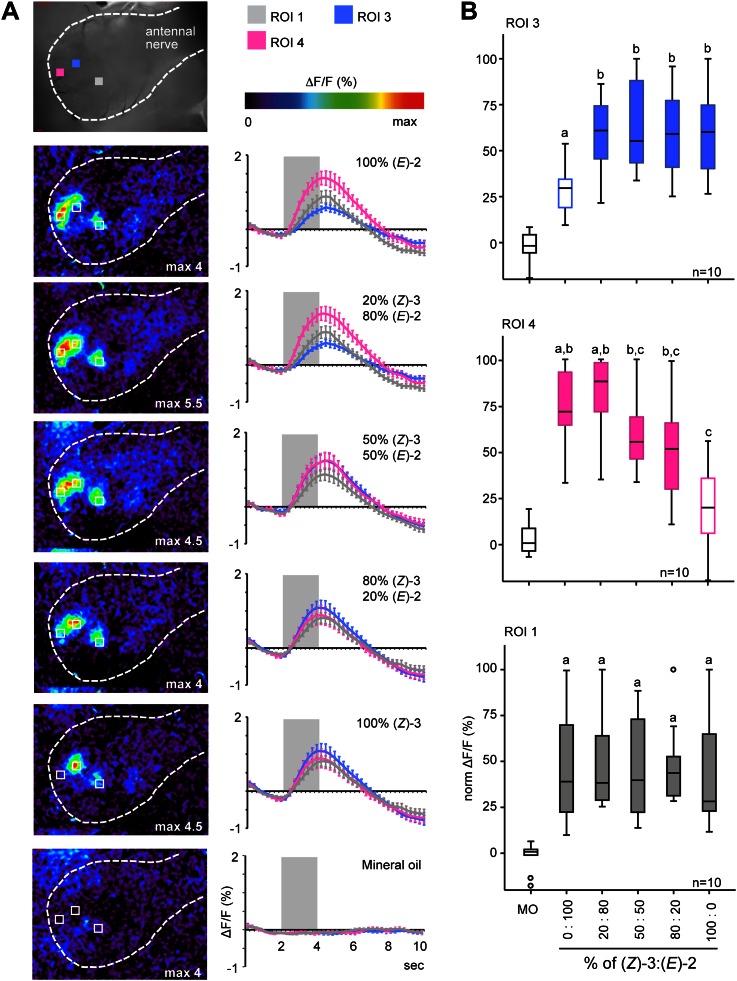
Female antennal lobe (AL) shows isomer-specific calcium responses to (*Z*)-3- and (*E*)-2-hexenyl acetate. (**A**) Representative false color-coded images show calcium responses in the AL after odor stimulation with isomeric mixtures of a total dose of 250 ng. Images are individually scaled to the strongest activation (given by the max value in each image). Time traces show activity of ROI 1, 3 and 4 (n = 10) in response to odor stimulation (2 s; grey bar). Error bars represent standard error of mean. For raw data, see F5A_AllmannSpaethe2012_timetraceshexenylacetate.xlsx (Dryad: Allmann et al., 2012). (**B**) Change in fluorescence in ROI 1, 3 and 4 to the pure structural isomers and their mixtures, normalized to the highest activation in every animal. Filled boxes represent responses significantly different from the mineral oil (MO) control; different letters denote significantly different calcium responses (Kruskal–Wallis and Dunn’s multiple comparison test). For raw data, see F5BCE_AllmannSpaethe2012_imaginghexenylacetate.xlsx (Dryad: Allmann et al., 2012). **DOI:** http://dx.doi.org/10.7554/eLife.00421.009

As plants do not emit isomerically pure odors but rather mixtures, we studied AL representation of the acetate structural isomers in more detail by stimulating the antenna with blends of (*Z*)-3- and (*E*)-2-hexenyl acetate in different ratios (given as Z/E: 100/0, 80/20, 50/50, 20/80, 0/100). In ROI 3 (blue) calcium signals evoked by (*Z*)-3-hexenyl acetate-containing mixtures were significantly higher compared with stimulations with pure (*E*)-2-hexenyl acetate, which in turn did not differ from the mineral oil control (Figure 5A,B). For the (*E*)-2-specific ROI 4 (pink) stimulation with pure (*Z*)-3-hexenyl acetate led to significantly lower calcium responses when compared with pure (*E*)-2-hexenyl acetate and the 20/80 ratio, but was not different from stimulation with mineral oil (Figure 5B). Calcium responses of the unspecific ROI 1 (in grey) did not differ between the structural isomers and their mixtures (Figure 5B).

**Figure 6.**
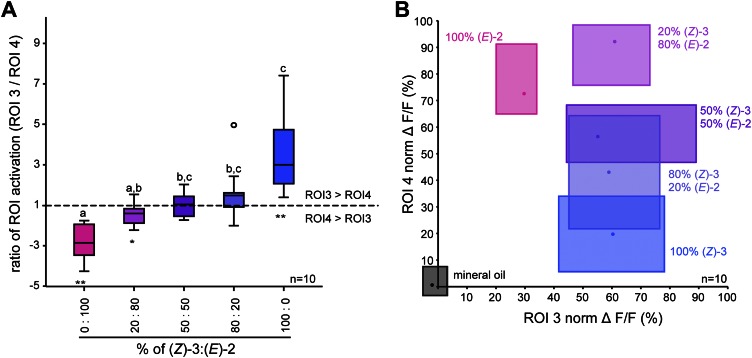
Isomer-specific regions show different response characteristics. (**A**) Both isomer-specific regions ROI 3 and ROI 4 are shown as ratios of ROI activation (ROI 3/ROI 4; for ROI 4 > ROI 3: −1/ratio) at stimulations with 250 ng. Asterisks indicate significant differences from 1, the ratio at which activation would be equal for ROI 3 and 4 (Wilcoxon signed-rank test, 100/0, 0/100: p<0.01, 20*/*80: p<0.05). Structural isomers and their mixtures were tested with Kruskal–Wallis and Dunn’s multiple comparison test, different letters denote significantly different ratios. (**B**) Calcium signals of ROI 3 (x-axis) and ROI 4 (y-axis) (% norm ΔF/F, separated by axes) in response to odor stimulation (colored boxes) and the solvent mineral oil (grey box). Points denote the median values, box outlines mark the 25% and 75% percentiles. For raw data, see F5BCE_AllmannSpaethe2012_imaginghexenylacetate.xlsx (Dryad: Allmann et al., 2012). **DOI:** http://dx.doi.org/10.7554/eLife.00421.010

When comparing odor-evoked activation by different (*Z*)-3/(*E*)-2-ratios in ROI 3 and 4, stimulations with pure structural isomers as well as the 20% (*Z*)-3/80% (*E*)-2 mixture led to significantly different levels of neural activity in these (*E*)-2/(*Z*)-3-specific regions (Figure 6A). Activation patterns differed significantly for pure (*E*)-2-hexenyl acetate compared with the 50/50 and 80/20 (*Z*)-3/(*E*)-2 mixtures as well as for pure (Z)-3-hexenyl acetate compared with the 20/80 mixture (Figure 6A). However, no differences were found between the isomeric mixtures (20/80; 50/50; 80/20).

In addition to the isomer-specificity for both hexenyl acetates, ROI 3 and 4 displayed different response characteristics (Figure 6B). The level of activation of the (*Z*)-3-hexenyl acetate-specific ROI 3 (x-axis) was solely dependent on the presence of the (*Z*)-3-isomer and did not change with various amounts of it in the isomeric mixtures (ranging from 50 ng in 20/80 to 250 ng in 100/0). In contrast, the calcium signal in ROI 4 (y-axis) increased gradually with increasing percentage of the (*E*)-2-isomer up to 80% in the isomeric mixtures. Thus, ROI 4 is able to convey information about the ratio of (*Z*)-3- and (*E*)-2-hexenyl acetate in a mixture.

### (*Z*)-3- and (*E*)-2-GLVs elicit different behavioral responses in ovipositing *Manduca* moths in nature

**Figure 7.**
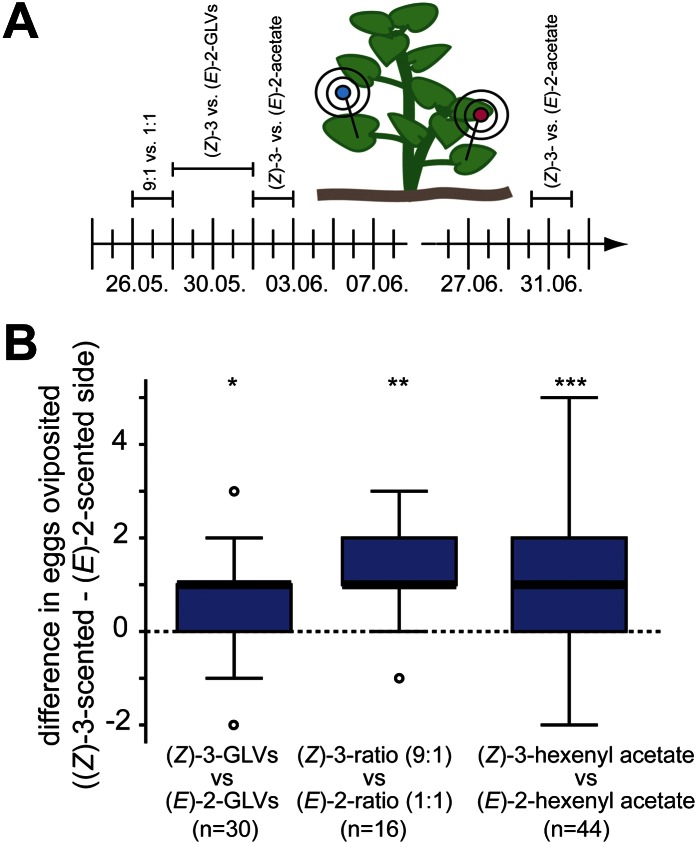
*Manduca* moths laid more eggs on the (*Z*)-3- than on the (*E*)-2-scented side of the plant. (**A**) The effect of different GLV-mixes on the oviposition behavior of female *Manduca* moths was tested during the 2010 field season on native *Datura wrightii* plants. On each experimental day, two different mixes were tested in a paired design. Mixes used on different experimental days are plotted above the timeline. The detailed composition of each mixture is described in Table 3. (**B**) Difference in number of eggs oviposited per plant. Higher oviposition rates were observed for the (*Z*)-3-scented side of the *D. wrightii* plants. Treatment pairs with no oviposited eggs were excluded prior to the statistical analysis (Wilcoxon signed-rank test). The median is represented as a line in each box, box outlines mark the 25% and 75% percentiles; outliers are depicted as circles (if value > 1.5× the interquartile range). For raw data, see F6B_AllmannSpaethe2012_oviposition.xlsx (Dryad: Allmann et al., 2012). **DOI:** http://dx.doi.org/10.7554/eLife.00421.011

To test whether female *Manduca* moths use the herbivory-specific shift in (*Z*)-3/(*E*)-2-ratio to choose appropriate host plants for their offspring, we performed oviposition assays in the field during the 2010 field season (Figure 7A). We selected two native populations of *D. wrightii* plants located close to the Lytle Preserve research station (Santa Clara, UT). On each experimental day we tested two mixes that contained either only (*Z*)-3 or only (*E*)-2-GLVs or both structural isomers but in different ratios. Since our calcium imaging data suggested that *M. sexta* possesses (*Z*)-3- and (*E*)-2-hexenyl acetate specific glomeruli (and thereby OSNs) we also tested these two compounds separately (Table 3 gives composition of each GLV-mixture). Experiments were done in a paired design (Figure 7A) to minimize the volatile ‘noise’ caused by, for example, different numbers of flowers, different grades of damage or different plant ages.

**Table 3.** GLV-mixtures used for oviposition assay in the field **DOI:** http://dx.doi.org/10.7554/eLife.00421.012

	Compounds (common names)	*(Z)-3*/*(E)-2*-mix 1:1; ≈ w + OS (µg/µl lanolin)	*(Z)-3*/*(E)-2*-mix 9:1; ≈ w + w (µg/µl lanolin)
*(Z)-3*-GLVs	*(Z)*-3-hexenal (50% in triacetin)	5.0	9.0
	*(Z)-*3-hexenol	5.0	9.0
	*(Z)*-3-hexenyl acetate	0.05	0.09
	*(Z)*-3-hexenyl butyrate	0.05	0.09
*(E)-2*-GLVs	*(E)*-2-hexenal	5.0	1.0
	*(E)*-2-hexenol	5.0	1.0
	*(E)*-2-hexenyl acetate	0.05	0.01
	*(E)*-2-hexenyl butyrate	0.05	0.01
	Triacetin per 10 mL mix (derived from *(Z)*-3-hexenal), μl	51.25	92.2
	Triacetin added per 10 mL mix, μl	40.95	0
	Total amount of triacetin per 10 mL mix, μl	92.2	92.2
	**Compounds (common names)**	**Only *(Z)*-3-mix (µg/µl lanolin)**	**Only *(E)*-2-mix (µg/µl lanolin)**
*(Z)*-3-GLVs	*(Z)*-3-hexenal (50% in triacetin)	10.0	0.0
	*(Z)-*3-hexenol	10.0	0.0
	*(Z)*-3-hexenyl acetate	0.1	0.0
	*(Z)*-3-hexenyl butyrate	0.1	0.0
*(E)*-2-GLVs	*(E)*-2-hexenal	0.0	10.0
	*(E)*-2-hexenol	0.0	10.0
	*(E)*-2-hexenyl acetate	0.0	0.1
	*(E)*-2-hexenyl butyrate	0.0	0.1
	Triacetin per 10 ml mix (derived from *(Z)*-3-hexenal), μl	102.5	0
	Triacetin added per 10 ml mix, μl	0	102.5
	Total amount of triacetin per 10 ml mix, μl	102.5	102.5
	**Compounds (common names)**	***(Z)*-3-hexenyl acetate (µg/µl lanolin)**	***(E)*-2-hexenyl acetate (µg/µl lanolin)**
	*(Z)*-3-hexenyl acetate	5.0	0.0
	*(E)*-2-hexenyl acetate	0.0	5.0

When plants were augmented with isomerically pure mixtures that consisted of either (*Z*)-3 or (*E*)-2-GLVs (aldehyde, alcohol, hexenyl acetate and hexenyl butyrate, Table 3) *Manduca* moths laid significantly more eggs on the (*Z*)-3 than on the (*E*)-2-scented side of the plant (mean ± SEM number of eggs oviposited per plant side: (*Z*)-3-isomers 1.0 ± 0.2, (*E*)-2-isomers 0.5 ± 0.1, Figure 7B). When we compared two GLV mixes that contained all tested (*Z*)-3 and (*E*)-2-GLVs in a balanced isomeric ratio (1:1) or in a high (*Z*)-3 vs (*E*)-2-ratio (9:1), significantly more eggs were oviposited on the sides of the plants that were scented with the higher (9:1) (*Z*)-3/(*E*)-2-ratio (9:1-ratio 1.8 ± 0.2; 1:1-ratio 0.6 ± 0.2; Figure 7B). Finally, when we compared the two different hexenyl acetates, on average one additional egg per plant was oviposited on sides scented with the (*Z*)-3-isomer ((*Z*)-3-hexenyl acetate 1.8 ± 0.3, (*E*)-2-hexenyl acetate 0.9 ± 0.1; Figure 7B).

## Discussion

Here we demonstrate that the (*Z*)-3/(*E*)-2-ratio of the GLV bouquet emitted from *D. wrightii* plants differs depending on the presence or absence of *M. sexta* larval oral secretions at sites of simulated feeding-damage. As OS-specific changes in the (*Z*)-3/(*E*)-2-ratio were detectable during one of the two nights in field experiments, this volatile signal may be encountered by ovipositing *Manduca* females searching for host plants. Functional imaging experiments revealed that *M. sexta* females detect (*Z*)-3- and (*E*)-2-hexenyl acetate with distinct OSN populations leading to discrete activation patterns in the AL. In field experiments *Manduca* females laid fewer eggs on plants scented with GLV mixtures with increased proportions of (*E*)-2-GLVs.

Our initial laboratory studies indicated that OS-induced changes in the GLV-profile of *Datura wrightii* plants are detectable during day and night, but they also revealed that light plays a role for the magnitude of this change in the signal. It has been shown that darkening can cause a temporary burst of GLVs in plants (Graus et al., 2004; Brilli et al., 2011). Furthermore, in *Nicotiana attenuata* the lipoxygenase Na*LOX2*, which specifically provides oxygenated fatty acids for the GLV-pathway, has its highest transcript levels during the night (Allmann and Baldwin, 2010), and, while this might explain the overall increase in GLVs with decreasing light intensities, it does not explain the specific increase in (*E*)-2-GLVs in w + w treated plants (Figure 3 and Table 1, night). (3*Z*):(2*E*)-enal isomerase activity has been found in crude extracts of some plants (alfalfa and soybean; Takamura and Gardner, 1996; Noordermeer et al., 1999), but not in *N. attenuata* (Allmann and Baldwin, 2010), and it needs to be determined whether *Datura* plants possess such an enzyme with a nocturnal peak activity. Circadian rhythm is also known to affect volatile emission (Goodspeed et al., 2012) and might therefore be another factor involved in the variation in GLV emission found between the light and dark period.

Most research on herbivore induced plant volatiles has been done in laboratory studies under controlled conditions (Hunter, 2002; Kigathi et al., 2009). While these studies provide useful information about the influence of single stress factors, they often fail to include biotic and abiotic stresses that influence volatile production under natural conditions (Kigathi et al., 2009). To evaluate the importance of these stresses, we repeated our trapping experiments in the field using native populations of *D. wrightii* plants.

Night-GLV emissions were measured at two different dates; while (*Z*)-3/(*E*)-2-ratios were the same in w + w and w + OS treated plants during the first experimental night, shortly after a new moon, they differed significantly during the full moon, the second experimental night. Although quantitative differences in light intensities between the two experimental nights were not detectable with the instruments available on site, they were obvious to the human eye. The releases of several volatile compounds are known to exhibit diurnal photoperiodicity in their quantitative but also qualitative emission patterns (Loughrin et al., 1994; Turlings et al., 1995). In cotton, acyclic terpenes like β-farnesene and β-ocimene were emitted in a diurnal fashion, while GLVs and few terpenes did not show such a clear diurnal pattern (Loughrin et al., 1994). Diurnal-rhythm-dependent emission has also been observed in *N. tabacum* after feeding by larvae of *Heliothis virescens*, *M. sexta* and *Helicoverpa zea,* as these plants released larger amounts of (*E*)-2-hexenal during the night and emitted other GLVs exclusively in the dark period (De Moraes et al., 2001). Experiments with lima beans revealed that leaves damaged during the scotophase responded with an almost immediate nocturnal emission of (*Z*)-3-hexenyl acetate, while the main emission of β-ocimene was delayed and peaked during the photophase (Arimura et al., 2008). These studies affirm that light plays an important regulatory role in volatile emissions. Due to our sample size, it remains to be shown by further experiments whether moonlight is sufficient to regulate volatile emissions.

The herbivore-induced volatile blend comprises several groups of compounds such as GLVs, terpenoids and/or aromatics, all of which have been shown to mediate plant-insect interactions (Mumm and Dicke, 2010). GLVs, which were investigated in the present study, seem to play an important role in volatile ‘communication’ as almost every green plant releases them upon various stress conditions. Furthermore, GLVs are released instantly from plant tissue upon damage, independent of the time of day (Turlings et al., 1995; D’Auria et al., 2007), while terpenoids are released with a delay (Kant et al., 2004) and several terpenoids not at all during the night as they are linked to photosynthesis (Arimura et al., 2008). This makes GLVs an important cue for ovipositing *Manduca* moths as they are active during sunset and night (Theobald et al., 2010) and thus need to rely on signals that are released during the scotophase.

The use of volatile blends for host location by insects depends heavily on the ability to detect and process olfactory signals. The insect’s olfactory system is highly sophisticated and enables detection of odors at very low concentrations (Hansson et al., 1999; Tanaka et al., 2009). However, in a redolent world, insects must distinguish host odors from a high background noise. Plant volatiles are detected by OSNs and these can be tuned to highly specific (Bruce and Pickett, 2011) or to ubiquitous host plant compounds (Hansson et al., 1999; Bruce et al., 2005). We found that stimulations with hexenal- and hexenol-structural isomers led to activation of a distinct region in the AL (ROI 2, Figure 4A,B). However, calcium signals evoked by (*E*)-2-GLVs were significantly stronger compared with those evoked by (*Z*)-3-GLVs (Figure 4B). This difference in activation intensity is likely a result from different binding affinities of the structural isomers to the olfactory receptor expressed by OSNs targeting the activated glomerulus (Hallem et al., 2004; Hooper et al., 2009).

Of all tested compounds, only the (*Z*)-3- and (*E*)-2-isomers of hexenyl acetate activated two different discrete regions in the AL of *M. sexta* females (Figures 4B,C and 5A,B) strongly suggesting different isomer-specific OSN populations on the insect antenna. This leads to the proposition that for *M. sexta* females, changes in the volatile emission of (*Z*)-3- and (*E*)-2-GLVs might primarily be detected via hexenyl acetate. Given that all other tested GLVs activated only ROI 2, the investment in isomer-specific receptors and consequently glomeruli to detect and process ubiquitous GLV compounds such as (*Z*)-3- and (*E*)-2-hexenyl acetate indicates the importance of the information content transferred by these compounds and their respective ratios. Specific responses to different types of green leaf volatiles have been reported both at physiological (Hansson et al., 1999; Røstelien et al., 2005) and behavioral levels (Reinecke et al., 2005).

For hexenyl acetate, AL activation patterns elicited by stimulations with mixtures of both structural isomers were purely additive suggesting no mixture interaction at the OSN and AL input levels, which is consistent with other studies (Deisig et al., 2006; Carlsson et al., 2007; Silbering and Galizia, 2007; Kuebler et al., 2012). When comparing the ratio of ROI activation, we did not find any difference between the mixtures (Figure 6A). This result is not surprising when taking the different response characteristics of ROI 3 and 4 into account. Calcium activity of ROI 4 in response to mixtures with increasing percentages of the (*E*)-2-isomer were dose-dependent, whereas the activation of ROI 3 to the same mixtures resembled more an ‘on–off’ mechanism and was thus solely dependent on the presence of the (*Z*)-3-isomer, leading to a constant bias towards (*Z*)-3-hexenyl acetate (Figures 5B and 6B). We can, however, not neglect the possibility that the logarithmic, dose-dependent phase in the neural dynamics of the neurons innervating ROI 3 lies at a concentration range below what was tested here.

The different response characteristics of ROI 3 and 4 might mirror the relevance of the odors for *M. sexta* females. (*Z*)-3-hexenyl acetate is a rather ubiquitously occurring plant volatile, which is released in large amounts after damage irrespective of its origin (Arimura et al., 2008; Mumm and Dicke, 2010). Electrophysiological experiments revealed that this compound elicited many responses in OSNs on the female *M. sexta* antenna: 60% of the tested sensilla (Spaethe et al., 2013) as well as 21 of 34 cells in the female AL (Kuebler et al., 2011) responded to this compound. (*E*)-2-hexenyl acetate, in contrast, has rarely been reported in insect-plant interactions (Whitman and Eller, 1990; Quiroz and Niemeyer, 1998; Williams et al., 2010), aside from its release among other GLVs after larval feeding of *M. sexta* on *N. attenuata* (Allmann and Baldwin, 2010) as an indication of actual larval damage. Thus, the presence of each structural isomer contains specific information but at different levels of resolution and in different contexts. In the case of (*Z*)-3-hexenyl acetate, the detected signal might also be relevant in long-range host location and host choice. Information about (*E*)-2-hexenyl acetate gained by ROI 4 in a dose-dependent fashion should, on the other hand, be most valuable at a short distance to the plant, possibly to locate the best spot for oviposition depending on the actual amounts emitted by different plant sites or to choose among neighboring plants with different levels of (conspecific) herbivory.

Numerous studies suggest that the ratio of plant volatiles is an important component of the olfactory signal (Visser and Avé, 1978; Bruce et al., 2009; Cha et al., 2011). Visser and Avé (1978) found several GLVs playing important roles in host recognition of *Leptinotarsa decemlineata*. Augmenting the volatile emission of a potato host plant with the single GLV components (*Z*)-3- or (*E*)-2-hexenol, (*E*)-2-hexanal or 1-hexanol resulted in a disruption of the orientation of *L. decemlineata* to the potato plant. Further studies found neurons specifically responding to these GLVs both at the periphery and in the AL of *L. decemlineata* (De Jong and Visser, 1988a, 1988b). In the case of the oriental fruit moth, *Grapholita molesta,* the ratio of a minor compound to the remaining components of a plant-derived synthetic mixture determined behavioral acceptance of this mixture, which could be associated with the response of two glomeruli in the female AL (Piñero et al., 2008; Najar-Rodriguez et al., 2010). We found that ovipositing *Manduca* moths distinguished between different (*Z*)-3/(*E*)-2-ratios and that they used these volatile cues to choose oviposition sites associated with less feeding competition and predation. However, independent of whether the mixtures tested were rather complex in their composition (9:1 vs 1:1 ratios), or less complex (only (*Z*)-3-GLVs vs only (*E*)-2-GLVs), or consisted of only a single compound ((*Z*)-3-hexenyl acetate vs (*E*)-2-hexenyl acetate) ovipositing *Manduca* moths continuously made a choice and always preferred the side of the plant that smelled more of (*Z*)-3-GLVs, or less of (*E*)-2-GLVs (Figure 7B). From our results we cannot conclude whether a complex GLV bouquet of different ratios provides more reliable information than do single compounds, but our results demonstrate that by adding a single component to the volatile bouquet of native *D. wrightii* plants one can alter the choice of ovipositing *Manduca* moths.

How did this behavior evolve? It has been shown that *M. sexta* moths learn to feed from flowers of non-hosts due to olfactory conditioning (Riffell et al., 2008, 2013). Experience could as well shape female oviposition choice as it has been shown in other moth species (Rietdorf and Steidle, 2002; Olsson et al., 2006). During oviposition *M. sexta* females might encounter the herbivory-specific signal, but can never experience the reward of oviposition success associated with it. A *M. sexta* larva feeding on plants, however, is continuously surrounded by GLVs emitted from wounded plants and more importantly encounters almost continuously a low (*Z*)-3/(*E*)-2-ratio caused by its own oral secretions. The retention of odor memory learned at the larval stage onto the adult stage has been shown to occur in *M. sexta* (Blackiston et al., 2008). However, in such a case you would rather expect a preference for OS-elicited bouquets, as the larva grew up in these. More experiments are needed to solve whether experience and learning are involved in the avoidance of herbivory-specific (*Z*)-3/(*E*)-2-ratios.

Almost every green plant releases volatiles in highly variable amounts and compositions. This makes it a challenge for host searching insects to simultaneously extract useful information while flying through the odor plumes from multiple sources. Our results show that the AL, the first odor processing center of the insect brain, has the capacity to resolve the composition of GLV blends as emitted by highly relevant host plants. Correspondingly, gravid females make an informed choice. They prefer oviposition sites with reduced predation and competition risks for their offspring, as indicated by the plant volatile bouquet. Future work will reveal whether increasing amounts of (*E*)-2-GLVs or rather changes in the (*Z*)-3/(*E*)-2-ratio at the background of other host odors provide crucial information for female *Manduca* moths.

## Material and methods

### Plant material and growing conditions

*Datura wrightii* seeds were initially purchased from B & T World Seeds (Paguignan, France) and subsequently harvested from plants propagated in the glasshouse. Plants were grown in 2 l pots in the glasshouse (23–25°C, 50–70% humidity, 16 hr light supplemented by Philips Sun-T Agro 400 W Na-vapor bulbs, 350–500 µmol/m^2^/s^1^ photosynthetic photon flux at plant level) and used for experiments 35–0 days after sowing.

For field experiments we used native populations of similar sized *D. wrightii* plants, which were located close to the Lytle Preserve research station. Wild plants at the field site and plants grown from purchased seeds showed high morphological similarity.

### Plant treatments

For all treatments, plants were wounded with a fabric pattern wheel to punch three rows of holes on each side of the midrib. Wounded leaves were immediately treated with 20 μl of deionized water (w + w) or with 1:3 (vol/vol) diluted *M. sexta* oral secretions (w + OS), which were pipetted directly onto the wounded leaf and gently dispersed across the surface. The OS was collected from third to fifth instar caterpillars which were fed on *D. wrightii* plants, and OS was stored under Argon at −20°C until usage.

### Volatile collection

Volatile collections were performed in a growth chamber (temperature 23–25°C, humidity 50–60%) on shelves equipped with diode arrays of white (approximately 420–690 nm), red (630–690 nm) and UV (380–420 nm). Diode arrays were programmed to simulate daylight, twilight and night conditions accordingly regarding both light intensity and spectral composition (16:8 hr light/dark cycle, Figure 2).

*D. wrightii* plants were placed in the chamber two days prior the experiment to acclimatize. On the experimental day 1 single leaf per plant was enclosed immediately after treatment between two 50-mL food-quality plastic containers (Huhtamaki, Bad Bertrich, Germany) secured with miniature claw-style hair clips. Ambient air was pulled through the collection chamber and a glass tube (ARS, Inc., Gainsville, FL; www.ars-fla.com) packed with glass wool and 20 mg of Super Q (Alltech, Düsseldorf, Germany; www.alltech.com). Airflow was created by a vacuum pump (model DAAV114-GB; Gast Mfg, Benton Harbour, MI; www.gastmfg.com) as described by Halitschke et al. (2000). For each time point and each treatment we trapped volatiles from five replicate plants. Directly after volatile sampling, we determined the fresh mass (FM) of each trapped leaf for further calculations.

In the field, we selected eight plants of approximately similar size for each measurement in a 10-m radius. For each plant, we estimated the total leaf damage and we counted the number of flowers. To account for differences in volatile emissions caused by different degrees of leaf damage we selected three equal sized leaves of each plant and randomly assigned each leaf to one of the treatments (control, w + w or w + OS). Each leaf was photographed to calculate the leaf area. We subsequently subtracted the amounts of volatiles emitted from untreated control leaves from those emitted from treated leaves of the same plant. We used a Li-COR Li-250A light meter with a Li-190SA quantum sensor (http://www.licor.com) to measure the photosynthetic active radiation during the different trapping periods. Weather data during the volatile collection were obtained from weather station KUTSTGEO6 located in St. George, UT (www.wunderground.com). The first two trappings were performed on the 3 and 4 of June, soon after new moon. During the day volatiles were sampled at an average light intensity of 1450 µmol/s/m^2^. During the night samplings, the light intensity was below the detection limit. The second trapping was performed in the night from the 14 to the 15 of June. Although it was a bright night (full moon) the average light intensity remained below the detection limit. On the experimental day, we enclosed single leaves directly after elicitation in polystyrene chambers fitted with holes at both ends. Air was pulled through the chamber and subsequently through a single-use charcoal trap (Orbo M32; Sigma-Aldrich, Seelze, Germany) as described in Kessler and Baldwin (2001). Charcoal traps were equipped with MnO_2_-coated copper gauze as ozone scrubbers (OBE Corporation, Fredericksburg, TX) to prevent oxidation of volatiles.

In all experiments, volatiles were trapped for 2 hr immediately after elicitation.

Both charcoal and SuperQ traps were eluted with 250 μl dichloromethane (DCM) into a GC vial after spiking each SuperQ trap with 320 ng and each charcoal trap with 240 ng tetralin (Sigma-Aldrich, Seelze, Germany) as an internal standard.

### Volatile analysis and quantification

Samples were analyzed on an Agilent 7890A gas chromatograph (Agilent Technologies, CA) with the injection port kept at 230°C, operated in split-less mode and connected to an Agilent 5975C mass spectrometer. One microliter of each sample was injected on a polar column (Innowax; 30 m, 0.25 mm ID, 0.25-µm film thickness; J&W Scientific, Folsom, CA) operated under a constant He flow of 1.1 ml/min. The GC oven was programmed to hold 40°C for 5 min, to increase the temperature at 5°C/min to 130°C, then increasing temperature at 30°C/min to a maximum of 240°C. The maximum temperature was held for 15 min. The transfer line to the MS was kept at 260°C. The MS was operated in electron impact mode (70 eV) with the ion source at 230°C and the quadrupole at 150°C. The detector monitored selected ions (SIM): hexenals: *m/z* 55, 69, 83; hexenols: *m/z* 55, 57, 67, 82; hexenyl acetates: *m/z* 67, 71, 82; tetralin: *m/z* 104, 132.

Retentions times for each GLV were ascertained using standards of (*Z*)-3-hexenal, (*E*)-2-hexenal, (*Z*)-3-hexenol, (*E*)-2-hexenol, (*Z*)-3-hexenyl acetate, (*E*)-2-hexenyl acetate, (Sigma-Aldrich, Seelze, Germany) and quantifications were done after normalization to the peak of IS tetralin with calibration curves for each compound (33, 10, 5, 1, 0.5, and 0.1 ng; n = 3 replicates) using single ion traces (hexenal *m/z* 83, hexenol and hexenyl actetate *m/z* 82). Emission rates were calculated based on fresh mass or surface area of the sampled leaves.

(*Z*)-3/(*E*)-2-ratios were calculated for each sample dividing the amount of the *(Z)*-3-GLV by the amount of its corresponding *(E)*-2-isomer. For visual simplifications (*Z*)-3/(*E*)-2-ratios <1 were depicted as their negative reciprocal.

### Insect rearing

*M. sexta* females were reared as described in Grosse-Wilde et al. (2011). Pupae were kept separately in paper bags at 25°C and 70% relative humidity under a 16:8 hr light/dark cycle. Naïve adult females were used in functional imaging experiments 2–4 days *post* emergence.

### Preparation and staining of adult females of *Manduca sexta*

Moths were restrained in 15-ml Falcon tubes with the head exposed and fixed with dental wax (Surgident; Heraeus Kulzer, Dormagen, Germany). The head capsule was opened and all tissues covering the antennal lobes were carefully removed. The brain was bathed with Calcium Green-2 AM (30 μmol; Invitrogen, Darmstadt, Germany, http://www.invitrogen.com) containing physiological saline solution (Christensen and Hildebrand, 1987) with 6% Pluronic F-127, (Invitrogen) for 90 min at 4°C. After staining the brain was rinsed several times with Ringer’s solution to remove remaining dye.

### Optical imaging of the antennal lobes

For imaging we used a Till Photonics imaging system (Martinsried, Germany) equipped with a CCD camera (Sensicam; PCO Imaging) connected to an upright microscope (Olympus BX51WI). Monochromatic excitation light was given at 475 nm (500 nm SP; Xenon arc lamp, Polychrome V) and fluorescence was detected with a LP515 emission filter and transmitted by a DCLP490 dicroic filter. The set-up was controlled by software Tillvision 4.0 (Till Photonics). Images were taken with a water immersion objective (Olympus, 10×/0.30). Four-fold symmetrical binning resulted in image sizes of 344 × 260 pixels with one pixel corresponding to an area of 2.5 × 2.5 µm (10× magnification).

### Odorants tested with optical imaging

Odors were chosen based on the results of a previous study (Allmann and Baldwin, 2010) and on volatile collections of *D. wrightii* plants performed for this study (Figure 1; (*Z*)-3- and (*E*)-2- hexenal, hexenol and hexenyl acetate [Sigma Aldrich, Seelze, Germany]). Odors were diluted in mineral oil and used in doses of 25, 250, and 2500 ng for the comparison of pure structural isomers. Hexenyl acetate was additionally tested as percentage mixtures of its (*Z*)-3- and (*E*)-2-isomers ranging from 0/100%, 20/80%, 50/50%, 80/20% to 100/0% (vol/vol) in doses of 250 and 1250 ng.

### Odorant stimulation

6 µl of the odorant mixtures were pipetted prior the experiment on a filter paper (Whatman, http://www.whatman.com/) in glass pipettes using doses of 25, 250, 1250 (isomeric mixtures) and 2500 ng, respectively. The same volume of mineral oil served as a control stimulus. The stimulus loaden pipette and a second empty pipette were inserted in parallel into a glass tube, which delivered a constant flow of clean humidified air (1 l/min) along one antenna. A continuous clean airstream (0.1 l/min) was directed through the empty pipette and switched to the odor-containing pipette (Syntech Stimulus Controller CS-55) during stimulation, thus preventing any change in total flow during the experiment.

Every stimulation experiment lasted for 10 s, recording 2 s pre- and 6 s post-stimulus and 2 s of odor stimulation. Inter-stimulus time of at least 1 min was chosen to reduce adaptation effects. Every odor was presented first in the lower concentration. The sequence of the stimulations was changed from animal to animal. In some females (hexenal n = 10, hexenol n = 14, hexenyl acetate n = 8), the odors were repeatedly measured to test for the reproducibility of the evoked activity patterns within an animal (Figure 4C).

### Processing of optical imaging data

All stimulation experiments were recorded with 4 Hz resulting in a series of 40 consecutive frames, which were analyzed with custom written software (IDL; ITT Visual Informations Solutions). Data were corrected for background fluorescence, bleaching of the dye and movement during the measurement (Sachse and Galizia, 2002, 2003). A spatial median filter of 5 pixels was applied to reduce shot noise.

Odor responses represented as change in fluorescence (ΔF/F) at spatially distinct activity spots were analyzed at the spot center in an area of the size of a small to medium-sized glomerulus (60 × 60 µm). Time traces of ΔF/F were smoothed by averaging three successive frames for each activity spot. The maximum ΔF/F value after stimulus onset was averaged with the pre- and postmaximum value. For every animal the odor responses were normalized to the maximal response and were taken into account if they reached ≥30% of the maximal value in this animal in at least one activity spot.

### Analysis of activity patterns in the moth antennal lobe

Due to the lack of a glomerular map in the *M. sexta* AL observed activity regions for the tested odors could not be directly compared between animals. Thus, activation patterns for every isomeric pair of hexenal, hexenol and hexenyl acetate were used to calculate correlation coefficients providing a relative measurement of similarity (Bisch-Knaden et al., 2012). Repeated stimulations with the same structural isomer and the correlation coefficients thereof were used as control.

To compare activity patterns between the different isomeric mixtures of hexenyl acetate we calculated the difference in activity of both isomer-specific glomeruli resulting from the ratio of activity in the (*Z*)-3-specific and the (*E*)-2-specific glomerulus. For visual simplifications values below 1 (representing cases in which the (*E*)-2-specific glomerulus was more active than the (*Z*)-3-specific glomerulus) were displayed as their negative reciprocal and all values were presented on a scale without the range between ‘−1’ and ‘1’ (Figure 6A).

### Oviposition assay in the field

Experiments were done between 26 May and 1 July 2010 in southwestern Utah. This area is part of the native habitat of the tobacco and tomato hawkmoths *Manduca sexta* and *M. quinqemaculata*. Eggs of both species were counted for this experiment. We selected between 15 and 17 plants of two native populations of *D. wrightii* plants, which were located close to the Lytle Preserve research station. All plants were carefully inspected and oviposited *Manduca* eggs were removed prior the experiment. On each experimental day two mixes were tested in a paired design: every evening before sunset (at around 5 pm) cotton swabs were dipped into the GLV-scented lanolin pastes and stuck onto two opposing branches of one *Datura* plant (Figure 7A). This paired design was chosen to minimize the effect that different numbers of flowers or different grades of leaf damage might have on the oviposition behavior of the moths. On the next day freshly laid *Manduca* eggs were counted in a defined area on the plant, approx 30 cm around the scented cotton swabs and afterwards removed. Treatment sides were switched every day. Plants with no oviposited eggs (isomers N = 36, ratios N = 15, acetates N = 18) were excluded prior to the statistical analysis. The GLV-scented lanolin pastes were prepared by warming up lanolin and adding different GLV-mixtures to the liquefied lanolin paste shortly before it solidified again. The GLV mixes used are described in Table 3. A comparison of emission rates and (*Z*)-3/(*E*)-2-ratios emitted from cotton swabs and w + OS and w + w treated *D. wrightii* plants is shown in Tables 4 and 5.

**Table 4.** Average (±SD) GLV emissions of GLV-mixtures used for the field bioassays (cotton swab, after Allmann and Baldwin, 2010) and of native *Datura wrightii* plants in the field during the first 2 hr after w + w or w + OS treatment; second night (0–2 am) **DOI:** http://dx.doi.org/10.7554/eLife.00421.013

Common names	Volatile release in µg/30 min			
	Cotton swab (after Allmann and Baldwin, 2010)		*D. wrightii* leaf	
	9:1 GLV mix	1:1 GLV mix	w+w	w+OS
(*Z*)-3-hexenol	9.8 ± 13.21	7.1 ± 9.71	0.20 ± 0.17	0.15 ± 0.07
(*Z*)-3-hexenyl acetate	0.18 ± 0.21	0.15 ± 0.18	0.24 ± 0.30	0.47 ± 0.67
(*E*)-2-hexenal	1.3 ± 2.14	4.3 ± 7.20	0.24 ± 0.27	0.48 ± 0.57
(*E*)-2hexenol	1.3 ± 1.84	8 ± 11.39	0.05 ± 0.06	0.07 ± 0.08
(*E*)-2-hexenyl acetate	0.06 ± 0.07	0.16 ± 0.20	0.04 ± 0.07	0.09 ± 0.14

**Table 5.** Average (*Z*)-3/(*E*)-2-ratios of GLV-mixtures used for the field bioassays (cotton swab, after Allmann and Baldwin, 2010) and of native *Datura wrightii* plants in the field during the first 2 hr after w+w or w+OS treatment; 2^nd^ night (0–2am) **DOI:** http://dx.doi.org/10.7554/eLife.00421.014

Common names	(*Z*)-3/(*E*)-2-ratio of emitted GLVs			
	Cotton swab (after Allmann and Baldwin, 2010)		*D. wrightii* leaf	
	9:1 GLV mix	1:1 GLV mix	w + w	w + OS
Hexenol	8.37	1.07	4.44	2.38
Hexenyl acetate	3.24	0.90	25.94	15.67

Emissions of *D. wrightii* were adjusted from leaf surface (cm^2^) to fresh mass (g) scale by the rough estimate of 50 cm^2^ = 1 g and represent the emission of two medium sized leaves.

**Table 6.** Comparison of trapping capability of adsorbents used in laboratory (SuperQ) and field volatile collections **DOI:** http://dx.doi.org/10.7554/eLife.00421.015

Compound name	Peak area × 10^6^ ± SD		
	RT	Activated charcoal	SuperQ
(*Z*)-3-hexenal	2.40	0.90 ± 0.13	5.4 ± 1.01
(*E*)-2-hexenal	3.57	9.3 ± 3.33	6.9 ± 1.21
(*Z*)-3-hexenol	3.35	10.1 ± 3.07	7.6 ± 1.49
(*E*)-2-hexenol		n.d.	n.d.
(*Z*)-3-hexenyl acetate	12.25	0.56 ± 0.28	0.47 ± 0.22
(*E*)-2-hexenyl acetate	12.68	0.52 ± 0.03	0.59 ± 0.04

Mean (±SD; n = 6) peak areas of GLVs emitted from *N. attenuata* plants in the glass house. Of each plant two equally sized leaves were mechanically wounded. Subsequently, volatiles were collected for 1 hr with traps filled with either SuperQ or activated charcoal. Traps were eluted with 250 μl Dichloromethane and measured on a GC-MS equipped with a BR-5ms column (Bruker, 15 m, 0.25 mm ID, 25 μm).

## Additional files

### Major datasets

The following dataset was generated:

AllmannS, SpätheA, Bisch-KnadenS, KallenbachM, ReineckeA, SachseS, BaldwinIT, HanssonBS, 2012, Data from: Feeding-induced rearrangement of green leaf volatiles reduces moth oviposition, http://dx.doi.org/10.5061/dryad.p7s88, Available at Dryad Digital Repository under a CC0 Public Domain Dedication.
