# Correction: Feeding-induced rearrangement of green leaf volatiles reduces moth oviposition

**DOI:** 10.7554/eLife.00992

**Published:** 2013-06-11

**Authors:** Silke Allmann, Anna Späthe, Sonja Bisch-Knaden, Mario Kallenbach, Andreas Reinecke, Silke Sachse, Ian T Baldwin, Bill S Hansson

**Keywords:** *Manduca sexta*, plant volatiles, oviposition, Ca imaging, *Datura wrightii*, Other

Allmann S, Späthe A, Bisch-Knaden S, Kallenbach M, Reinecke A, Sachse S, Baldwin IT, Hansson BS. 2013. Feeding-induced rearrangement of green leaf volatiles reduces moth oviposition. *eLife* **2**:e00421. doi: http://dx.doi.org/10.7554/eLife.00421. Published 14 May 2013

The first sentence of paragraph 2 and the first sentence of paragraph 4 of the eLife digest (doi: http://dx.doi.org/10.7554/eLife.00421.002) incorrectly gave the trivial name of *Datura wrightii* as ‘devil’s claw’. This has been corrected to ‘sacred Datura’.

The article has been corrected accordingly.

