# Supplementary material for: Feeding-induced rearrangement of green leaf volatiles reduces moth oviposition
Source: eLife. 2013 May 14;2:e00421. doi: 10.7554/eLife.00421 (PMC3654435; doi:10.7554/eLife.00421)
Supplement: Related texts

# Article commentary: Decision letter

**DOI:** 10.7554/eLife.00421.016

**Affiliations:** Wageningen University, The Netherlands

eLife posts the editorial decision letter and author response on a selection of the published articles (subject to the approval of the authors). An edited version of the letter sent to the authors after peer review is shown, indicating the substantive concerns or comments; minor concerns are not usually shown. Reviewers have the opportunity to discuss the decision before the letter is sent (see review process). Similarly, the author response typically shows only responses to the major concerns raised by the reviewers.

Thank you for choosing to send your work entitled “Small changes, large consequences – feeding-induced rearrangement of green leaf volatiles reduces moth oviposition” for consideration at *eLife*. Your article has been evaluated by a Senior editor, Detlef Weigel, and 3 reviewers, one of whom, Marcel Dicke, is a member of our Board of Reviewing Editors.

The Reviewing editor and the other reviewers discussed their comments before we reached this decision, and the Reviewing editor has assembled the following comments to help you prepare a revised submission.

The effect of herbivory on plant volatile emission has been investigated for some 30 years, and green leaf volatiles have been the subject of many studies. Recently, Allmann & Baldwin showed that *Manduca* oral secretions alter the Z/E ratio of green leaf volatiles in *Nicotiana attenuata*, with a shift towards E isomers. The present manuscript merges Baldwin’s field program in which wild hawkmoths are major selective agents on *Nicotiana attenuata* plants, with Hansson’s lab-oriented program of using *Manduca sexta* as a model system for olfactory coding, perception, and behaviour. This is an exciting prospect for chemical ecologists, synergizing the potential of these model systems in the field. The premise is that a subtle shift in stereochemistry provides information for the insects (friends and foes alike) interacting with wounded/induced plants.

All three reviewers find the study interesting, especially because of the combination of methodologies used. The study is a valuable follow-up of the paper by Allmann and Baldwin in *Science* that provides information on the perception of the GLVs by *Manduca sexta* females and behavioural choices under field conditions by females of two *Manduca* species.

However, the reviewers also identify several important issues to be addressed before this manuscript can be accepted for publication by *eLife*. The major issues are:

1) Explaining the rationale of the study and the arguments for setting up this study, such as the connection to the 2010 *Science* paper by Allmann and Baldwin, as well as linking the study better to the study by Visser and Ave in 1978 and subsequent work by JH Visser & R De Jong that also concern neurobiological aspects and behavioural studies of the role of individual GLVs in a total blend.

2) The aspect of light, which is not well introduced and sounds like a post-hoc aspect, which in connection with the fact that there is only a single replicate for the silver moon and full moon weakens the conclusions rather than strengthening them. You may consider eliminating this aspect altogether.

3) The ecological relevance of the study (origin of seeds, combining two different moth species, comparison with plants with feeding damage in the field).

4) Explaining methodological aspects, with a focus on those aspects that may have affected the conclusions (e.g., different adsorbents for lab and field experiments, and differences in headspace composition), and on the choice for the methods used while not using other methods, such as peripheral recordings or controls such as sham control plants.

# Reply: Author response

**DOI:** 10.7554/eLife.00421.017

*1) Explaining the rationale of the study and the arguments for setting up this study, such as the connection to the 2010 Science paper by Allmann and Baldwin, as well as linking the study better to the study by Visser and Ave in 1978 and subsequent work by J.H. Visser & R. De Jong that also concern neurobiological aspects and behavioural studies of the role of individual GLVs in a total blend*.

While our study and results are very much placed in an ecologically relevant background, the studies of Visser and colleagues covered fundamentals in host recognition and odor processing. Of course, there are some parallels between these classical studies and our experiments, like the focus on GLV compounds; however, the underlying questions differ fundamentally. Thus, care must be taken when comparing our results with those of Visser & de Jong. We were asking whether *M. sexta* is able to differentiate between wounding signals and detect the difference between feeding-damaged and mechanically wounded plants via different ratios of (*E*)-2-and (*Z*)-3- GLVs. We did not apply an arbitrary manipulation to the host plant, but an ecological relevant and naturally occurring variation in the volatile blend of a host plant. We did not compare intact with manipulated plants; we used a paired design testing two different variations of volatile blends of damaged host plants.

The pioneering studies by Visser & Ave and Visser & de Jong focused on general principles in host recognition and ratio-dependent odor processing of GLV blends. They showed how ratios of volatiles are used by the olfactory system for host recognition. Intracellular recordings of OSNs revealed differences in the coding of single GLVs and binary mixes, and highlighted the role of suppression during odour processing.

We revised the Introduction and Discussion to clarify the motivation of the study, and to incorporate the work of Visser & de Jong & colleagues.

*2) The aspect of light, which is not well introduced and sounds like a post-hoc aspect, which in connection with the fact that there is only a single replicate for the silver moon and full moon weakens the conclusions rather than strengthening them. You may consider eliminating this aspect altogether*.

We are convinced that the light regime is a very strong candidate to explain the variation in our data. We admit that the small sample size is unsatisfactory and more experiments will be needed to shed more “light” onto the effect of light on GLV emissions. However, we feel that these experiments are beyond the scope of our present manuscript. We now emphasize the possible effect of light in our Introduction to avoid post-hoc concerns.

*3) The ecological relevance of the study (origin of seeds, combining two different moth species, comparison with plants with feeding damage in the field)*.

We agree that it would have been better to work with *D. wrightii* seeds from the field site in Utah. However, the collection of wild seeds at our field site during the last 3 years was impeded by a greatly expanded rodent population tracking the brome grass invasion. The animals showed exceptional seed eating behavior, making wild seeds mostly unavailable. Thus, we had to search for alternatives. Unfortunately, B&T World Seeds do not indicate a clear origin of their *D. wrightii* seeds. However, plants grown from purchased seeds phenotypically matched those grown from the few available wild seeds in the glass house. We have added this information to the manuscript.

We are convinced that differences between plant seed origins are not the cause of our variation in the emission of (*E*)-2- and (*Z*)-3-GLVs (Allmann and Baldwin, 2010). The elicitor of the altered (Z)/(E)-ratio is in the oral secretions of M. sexta larvae. Consequently, as long as the plants respond with the release of GLVs, the expected shift in the (*Z*)/(*E*)-ratio should occur. Hare (2007) reported differences in emission of induced volatiles between glandular and non-glandular phenotypes of *D. wrightii*. He, however, collected volatiles 48 h after damage and focused on the emission of sesquiterpenes. In the only assessed GLV, (*Z*)-3-hexenyl acetate, no difference was observed as the (*Z*)/(*E*)-ratio is in the oral secretions of *M. sexta* larvae. Since we were interested in the GLV emission, we collected the treated leaves immediately after they were damaged, which is the ecologically relevant time to do so, as this GLV emission provides ovipositing hawkmoths with accurate information about intraspecific competition and potential predation risk for their offspring.

Concerning the two moth species, we admit that we could not assign the deposited eggs to one single species with certainty. In the field experiments it is not possible to separate the two *Manduca* species from each other; both moths fly at the same time and their eggs look exactly the same.

However, our behavioral data show that whichever species oviposited on our experimental plants, they clearly differentiated between both treatments. Thus, we can conclude that either only *M. sexta* or both species use the (*Z*)/(*E*)-GLVs as information to choose appropriate host plants. Controlled laboratory experiments with each species could unambiguously reveal whether both species use the signal. However, it is extremely difficult to rear *Manduca quinquemaculata* and controlled greenhouse/growth chamber experiments with moths do not always produce the same results found in field experiments (Reisenman et al., 2013; Spaethe et al., 2013).

The integration of a treatment applying *M. sexta* larval feeding to *D. wrightii* plants in the field would very nicely complement our dataset, but under field conditions this is extremely difficult to establish. The occurrence of ovipositing moths is barely predictable both in time and location. To compare the treatments on one plant, the feeding larvae would need to be restrained to a certain area to allow the evaluation of a distinct radius. Furthermore, the number of larvae previously reared on *D. wrightii* needed for this experiment would probably exceed the scope of the field station.

*4) Explaining methodological aspects, with a focus on those aspects that may have affected the conclusions (e.g., different adsorbents for lab and field experiments, and differences in headspace composition), and on the choice for the methods used while not using other methods, such as peripheral recordings or controls such as sham control plants*.

The use of volatile traps with different adsorbents is a concession to the challenging group-organized fieldwork conditions and economic factors. Throughout the short but experimentally dense field season we need hundreds of traps for numerous experiments. Since these traps are stored at −20°C at the field location and transported to the laboratory before elution and analysis, it is organizationally and economically not possible to use the costly reusable traps filled with SuperQ that we use in the lab.

The absence of (*Z*)-3-hexenal in our field samples is most probably associated with the unstable nature of this compound. Volatile collections using active charcoal are not the cause for this previously reported variation between field and lab volatile collections. We attach new data showing that under laboratory conditions, both SuperQ and activated charcoal traps are capable of collecting (*Z*)-3-hexenal (Table 6).

Concerning the physiological experiments, we were asking whether *M. sexta* females are able to differentiate between wound signals and detect the difference between feeding-damaged and mechanically wounded plants. This ability necessitates specific OSNs to distinguish between the emitted (*E*)-2- and (*Z*)-3-conformers. We employed functional imaging to investigate the spatial patterns of glomerular activation in the antennal lobe. This technique is very powerful as it visualizes the complete input to the antennal lobe, i.e., the responses of thousands of OSNs at the same time. Due to the principles of OSN targeting, i.e., that OSNs expressing the same receptor type innervate one and the same glomerulus (Gao et al., 2000; Vosshall, 2000), the Ca^2+^-imaging technique reveals the involvement of different OSN types in an odor response. Electrophysiological experiments like single-sensillum or intracellular recordings show responses of single neurons and can provide information about information detection and processing via their response characteristics. This, however, was not the focus of our study. Since the *Manduca* AL receives input from roughly 250,000 OSNs from more than 20,000 sensilla, the search for specific OSN types by peripheral recordings would result in hitting neurons by chance to find differentially responding cells. Imaging provides the best of two worlds: specificity and completeness. Instead of registering the activity of single neurons, the specific response of the complete OSN population can be observed after a single odor stimulation.

Regarding sham controls, we were asking whether Manduca moths can distinguish between a damaged and a feeding-damaged plant, which were represented by two different ratios of (*Z*)-3- and (*E*)-2-GLVs. We focused on the sophisticated evaluation of host quality as it is likely to occur in nature, where plants are rarely free of damage or herbivory. We did not perform any sham controls, because they are not the focus of our study. One can speculate that a sham control would have received considerably more eggs than our treated plants, but how would that relate to the scientific question?
